# The combined application of rutin and silicon alleviates osmotic stress in maize seedlings by triggering accumulation of osmolytes and antioxidants’ defense mechanisms

**DOI:** 10.1007/s12298-024-01430-z

**Published:** 2024-03-09

**Authors:** Namuun Altansambar, Asiye Sezgin Muslu, Asim Kadıoglu

**Affiliations:** Department of Biology, Faculty of Science, Karadeniz Technical University, 61080 Trabzon, Turkey

**Keywords:** Rutin, Silicon, Antioxidants, Osmolytes, Gene expression

## Abstract

Silicon (Si) has been shown to improve plant defenses against a variety of stresses. However, how rutin (Rut) affects stress factors is yet to be fully explored. Moreover, their combined role in osmotic stress response remains unclear. The current study was performed to determine how the use of Rut and Si, both separately and in combination, improved the physiological resilience of maize seedlings to two levels of osmotic stress (induced by polyethylene glycol (PEG) 6000). We aimed to enhance osmotic stress tolerance with the simultaneous use of Rut and Si. First, we selected the best water status and the lowest membrane damage enhancing concentration of Rut (60 ppm) and Si (1 mM) to research their tolerance and resistance to osmotic stress (moderate: 10% PEG, severe: 15% PEG). The application of Rut and Si separately and together reduced oxidative stress by decreasing the reactive oxygen species and improved the relative water content, osmoprotectants (proline, total soluble sugar, and glycine-betaine), ascorbate level, and some antioxidant defense-related enzyme activities and their gene expression in maize seedlings under osmotic stress. However, these effects were more promising under moderate stress. As a result, findings from the study indicate the synergistic effect of combined Rut and Si on osmotic stress tolerance in maize seedlings. Overall, the combination of Rut and Si was more effective than independent Rut and Si in reducing osmotic stress in maize seedlings. Here, it was clear that Rut played an active role in alleviating stress. This combined application can be useful for developing drought tolerance in crops for the agriculture sector.

## Introduction

Drought stress reduces membrane stability, water content, protein content, and photosynthetic activity, all of which lead to lipid peroxidation, membrane damage, and increased generation of reactive oxygen species (ROS) in plants (Naz et al. 2021; Hanafy and Sadak 2023). In response to drought stress, plants boost the synthesis of phytohormones, osmoprotectants, and antioxidant enzymes (Azmat et al. 2020; El-Bassiouny et al. 2023). Drought-related yield losses in cereal crops vary greatly across countries (Zulkiffal et al. 2021; Sadak 2022). Metabolites should be used as foliar or root applications and seed priming agents to improve plant tolerance or resistance to stress factors (Riffat and Ahmad 2020; Konakçı et al. 2020; Maqsood et al. 2021Sadak and Bakhoum, 2022).

Silicon (Si) is a plentiful element that is beneficial in several ways to plant growth and development, especially in stressful conditions (Zargar et al. 2019). Significant variability in Si accumulation among plant species has been reported (Deshmukh et al. 2020). Si influences crop quality, plant development, yield, and drought resistance or tolerance in plants (Ahanger et al. 2020). Furthermore, Si can improve disease resistance by interacting with a variety of key chemicals involved in plant stress signaling (Rodrigues et al. 2004). Si has also been shown to increase osmoregulation, leaf water content, and the photosynthetic rate in plants, all of which can improve drought stress tolerance (Luyckx et al. 2017; Tripathi et al. 2020).

Rutin (Rut), a flavonoid phenolic compound found in plants such as asparagus (Wang et al. 2003), has antioxidant properties and has been shown to reduce lipid peroxidation (Yang et al. 2008). Compared to other antioxidant compounds, few studies have been undertaken to investigate the impact of Rut on plants growing under stress conditions (Ferdinando et al. 2012; Ismail et al. 2015; Singh et al. 2017; Yang et al. 2022). It was observed that the amount of Rut and other phenols increased in *Hypericum brasiliense* and *Artemisia* plants exposed to drought stress (Kumar et al. 2021). Overexpression of the drought stress tolerance gene (*NtCHS*) in the drought-resistant plant *Nicotiana tabacum* was found to increase drought stress tolerance by stimulating the accumulation of Rut and other flavonoids (Hu et al. 2020).

To the best of our knowledge, whether exogenous Rut has utility in protecting plants from stress factors is yet to be fully determined. Additionally, there is a scarcity of reports that show the effects of individual and/or combined Rut and Si application on osmotic stress. Therefore, in the current study, it was hypothesized that (1) Rut and Si would show a synergistic effect against osmotic stress in maize seedlings, (2) This effect would achieve drought tolerance by maintaining water status and modulating the antioxidant defense and osmotic adjustment systems of maize seedlings. Our study will provide new insights into elucidating the physiological and molecular mechanisms of Rut and Si in maize seedlings' responses to osmotic stress.

## Material and methods

### Plant material, experimental design, and applications

*Zea mays* L. was cultivated hydroponically in a growth chamber with Hoagland’s solution (Hoagland and Arnon 1950). The growth conditions were as follows: humidity 60 ± 4%, day/night temperatures 25/22 °C, and PPFD 400 µmol m-^2^ s^−1^. After 21 days of growth, Rut (30, 60, and 90 ppm) and Si (0.5, 1, and 2 mM) were applied to the seedlings. After 24 h, the seedlings were exposed to osmotic stress (10% and 15% polyethylene glycol (PEG_6000_)) in Hoagland’s solution for 72 h. Si was added in the form of CaSiO_3_. Seedlings treated without PEG were used as the control group. It was determined that under osmotic stress (10% and 15% PEG) conditions plants treated with 60 ppm Rut and 1 mM Si had better water status and lower membrane damage. Therefore, these concentrations were used in the experiments. The experimental pots were subjected to nine different treatments: (1) the nutrient solution as the control treatment, (2) moderate stress: 10PEG, (3) severe stress: 15PEG, (4) Rut + 10PEG, (5) Si + 10PEG, (6) Rut + Si + 10PEG, (7) Rut + 15PEG, (8) Si + 15PEG, (9) Rut + Si + 15PEG. When the applications were completed, 25-day-old seedlings were harvested and tested in terms of the following parameters.

### Leaf relative water content (RWC)

Approximately 0.1 g (fresh weight (FW)) of maize leaves were initially weighed using a balance. Subsequently, these leaves were immersed in water and rehydrated for 24 h at 4 °C in darkness to determine the water-saturated weight (SW). The dry weight (DW) of the leaves was obtained by keeping them in an oven at 75 °C for 48 h. The RWC of the leaves was calculated using the following formula: RWC (%) = (FW − DW)/(SW − DW) × 100 (Castillo 1996).

### Thiobarbituric acid reactive substances (TBARS) content assay

A 0.1-g leaf sample was thoroughly mixed with 1.8 mL of 0.1% trichloroacetic acid (TCA) using a tissue homogenizer. Subsequent to centrifugation at 16,000 × *g* for 5 min, 4 mL of 0.5% thiobarbituric acid, prepared using 20% TCA, was introduced to 1 mL of the resulting supernatant. This mixture was then kept in an oven set to 95 °C for 30 min. Following cooling to room temperature, the absorbance of the supernatant was measured at 532 nm, excluding nonspecific absorption readings at 600 nm from the calculations. The determination of TBARS content was performed utilizing the formula established by Heath and Packer (1968): ΔAbs (Abs532-Abs600) = Ɛ.C.L (Ɛ = 155 mM^−1^ cm^−1^, L = 1 cm, C = mM, TBARS).

### Hydrogen peroxide (H_2_O_2_) content assay

The leaves (0.1 g) were homogenized with 1.8 mL of TCA (0.1%) and centrifuged. After that, potassium phosphate buffer (10 mM) and potassium iodide (1 mM) were added to the supernatant. At 390 nm, the absorbance was measured (Velikova et al. 2000).

### Osmolyte contents assay

To determine proline content, dried samples (0.1 g) were homogenized using 3% sulfosalicylic acid and centrifuged at 5000 × *g* for 5 min. Acetic acid and ninhydrin were added to the supernatant. The mixture was then placed in tubes at 100 °C in a water bath. To the cooled mixture, toluene was added followed by vortexing. The mixture was placed in capped tubes and centrifuged at 5000 × *g*. The upper phase was pipetted into a cuvette and measured at 520 nm (Bates et al. 1973).

In order to determine the total soluble sugar content, after homogenizing the dry leaf samples (0.1 g) with ethanol (70%) the homogenate was boiled at 80 °C. The homogenate was left to cool before being centrifuged at 10,000 × *g*. Purified water was mixed with the supernatant and diluted. This mixture was mixed with a stirrer after phenol (5%) was added. Sulfuric acid was added to the same mixture. The absorbance values of the reaction tubes were measured at 490 nm after they had cooled to room temperature (Dubois 1956).

To quantify the glycine betaine content, the deionized water was used to shake the extract prepared from 0.1 g of ground dry material. Subsequently, the samples underwent filtration. The filtrate was diluted with 2 N sulfuric acid. The measured liquid in the test tubes was cooled in ice water for 1 h. The mixture was gently vortexed after the addition of a cold reagent composed of potassium iodide and iodine. The samples were incubated for 16 h at 4 °C. Then the samples were centrifuged at 10,000 × *g* for 15 min. The supernatant was aspirated using a micropipette and dissolved in 1,2-dichloro ethane. After 2.5 h, absorbance was measured at 365 nm (Greive and Grattan 1983).

### Antioxidant defense system enzymes assay

Plant tissues (0.1 g) were finely crushed and subjected to extraction in 1.8 mL of extraction buffer (composed of K_2_HPO_4_, EDTA, PVPP, pH 7.0). Following centrifugation at 18,000 × *g* for 15 min, the resulting supernatant was employed for enzyme activity determination. Superoxide dismutase activity was assessed using the Beauchamp and Fridovich method (1971). The reaction commenced with the addition of 2 M riboflavin to a reaction medium containing 50 mM potassium phosphate buffer (pH 7.8), 0.1 mM EDTA, 13 mM L-methionine, 75 mM nitro blue tetrazolium, and 50 μL of extract. Absorbance values at 560 nm were measured after exposing the mixture to white light at 375 μmol m^−2^ s^−1^ for 10 min. Catalase activity was determined according to Aebi's method (1983), measuring absorbance at 240 nm for 5 min in a 1 mM reaction mixture consisting of 50 mM potassium phosphate buffer (pH 7.0), 30 mM H_2_O_2_, and 20 μL of enzyme extract. Ascorbate peroxidase activity was determined by monitoring the decrease in absorbance at 290 nm, following Nakano and Asada's method (1981). The reaction mixture (1 mL) included 50 mM potassium phosphate buffer (pH 7.0), 250 M ascorbate, 5 mM H_2_O_2_, and 20 μL of enzyme extract. For guaiacol peroxidase activity, the procedure outlined by Urbanek et al. (1991) was employed. Absorbance was measured at 470 nm for 1 min in a reaction mixture containing 100 mM potassium phosphate buffer (pH 7.0), 0.1 mM EDTA, 5 mM guaiacol, 15 mM H_2_O_2_, and 50 μL of enzyme extract. The glutathione reductase activity was measured spectrophotometrically as described by Foyer and Halliwell (1976). The assessment was based on the decrease in NADPH oxidation measured in absorbance at 340 nm for 5 min. Protein content was evaluated spectrophotometrically using the Bradford method (1976) to calculate enzyme activities.

### Ascorbate (AsA) content assay

The leaf samples (0.1 g) underwent homogenization with 5% m-phosphoric acid. After homogenization, the mixture underwent centrifugation at 10,000 × *g* for 4 min. The resultant material was then combined with citrate–phosphate buffer (pH 6.2) in the reaction medium. The initial absorbance was recorded at 265 nm, and the AsA content was determined by measuring the decrease 5 min after the addition of ascorbate oxidase to the reaction media. Once the ascorbate oxidase reaction was completed, it was inhibited using sodium azide. Subsequently, dithiothreitol (DTT) was added to the mixture, and after 3-min reduction with DTT, the absorbance was measured again at 265 nm (Liso et al. 1984).

### Antioxidant enzymes gene expression analysis

Total RNA was isolated using the Total RNA Isolation Kit (Favorgen FavorPrep Plant Total RNA Mini Kit). The quantity and purity of RNA samples were determined using a nanodrop spectrophotometer. For the cDNA synthesis, an Applied cDNA synthesis kit was employed. The resultant cDNAs were used in real-time PCR experiments to evaluate gene expression. The examination involved the use of 5 × HOT FIREPol Evagreen qPCR Supermix from Solis Biodyne and the CFX Connect Real-Time PCR System by BioRad. The RT-PCR method steps were modified using Solis Biodyne guidelines (Sezgin Muslu and Kadioglu, 2021). Each biological replication underwent analysis through three technical replicates, with an accepted mean technical error of 0.5 (± 1) Cq values. To assess the expression levels of *SOD*, *CAT*, *APX*, and *glutathione peroxidase* (Table 1), gene-specific primers were employed. The control utilized was the Actin gene. Gene expression levels were determined employing the 2^−∆∆Ct^ method, following the protocol outlined by Bookout and Mangelsdorf (2003).

**Table 1:** The sequences of specific primers used for qRT-PCR analysis

Target gene	Oligo name	Sequences 5’-3’
Actin (ACT)	ZmACT_F ZmACT_R	GAAGATCACCCTGTGCTGCT ACCAGTTGTTCGCCCACTAG
Superoxide dismutase (SOD)	ZmSOD_F ZmSOD_R	TTGTTGCAAATGCTGAGGGC AGGCAAGGATGTAACAGCGT
Catalase (CAT)	ZmCAT_F ZmCAT_R	TGCTTTCTGCCCAGCGATTA CACTTCTCACGACAGCCTGT
Ascorbate peroxidase (APX)	ZmAPX_F ZmAPX_R	GCCTTCTTCAGCTCCCAAGT TGCAAAAGACCACATGCAGC
Glutathione Peroxidase (GPX)	ZmGPX_F ZmGPX_R	CACGACTTCACCGTCAAGGA ACTGGGCAATCTCCTCGTTG

### Statistical analysis

All analyses were carried out with three replicates. The data of the experiments were expressed as the means ± standard error. Statistical analyses were performed using SPSS, a licensed software package based on Windows (version IBM 23), with two-way ANOVA. Pearson’s correlation analysis was performed to reveal the relationship between all the parameters.

## Results

### Leaf RWC

**Fig. 1:**
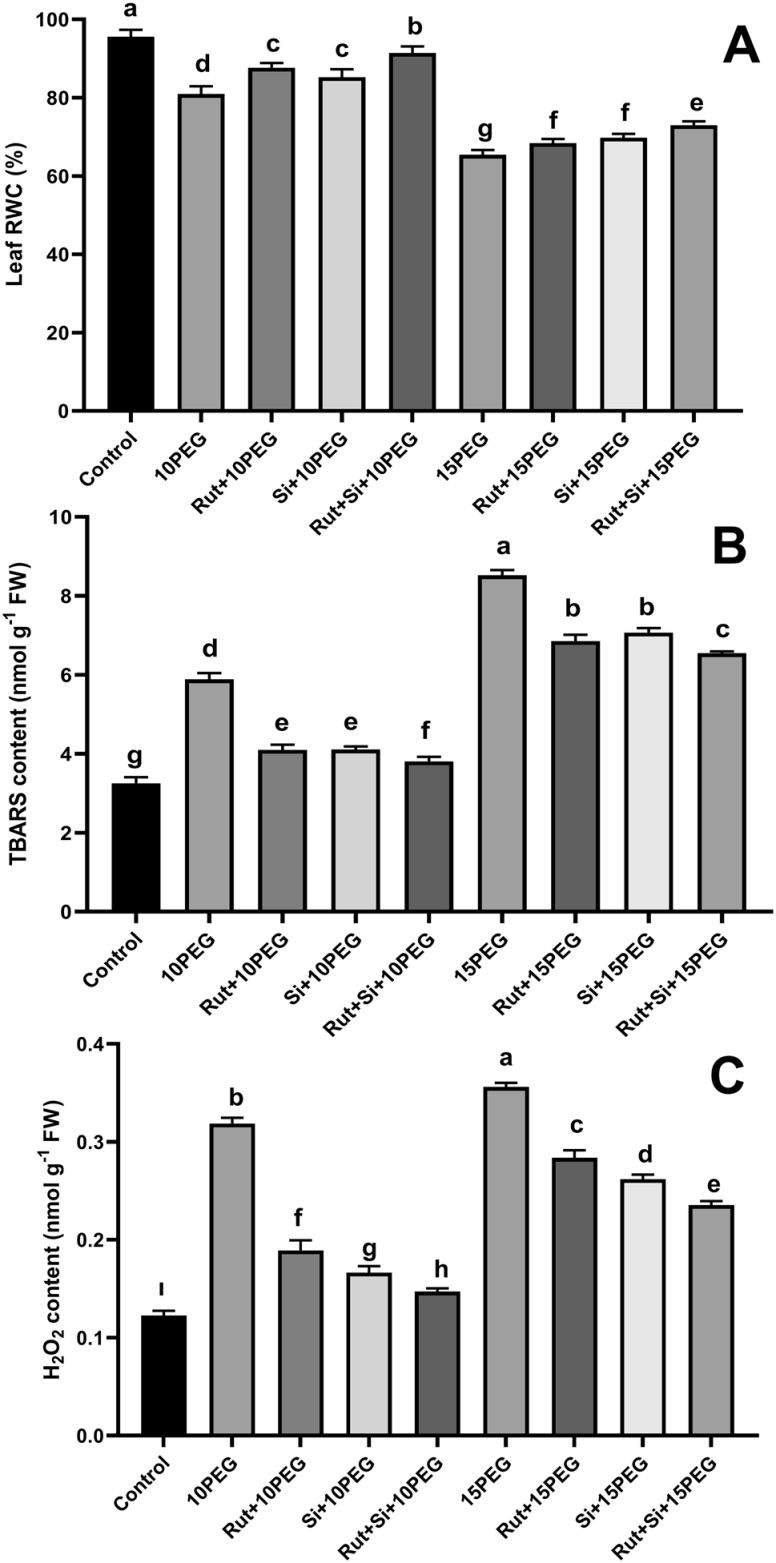
Effect of Rut and Si on Leaf RWC (**A**), TBARS level (**B**), H_2_O_2_ level (**C**) of maize seedlings under osmotic stress. Vertical bars represent standard deviations. Columns sharing the same letter indicate no significant difference at the 5% level

Leaf RWC was reduced in the seedlings under osmotic stress (exposed to 10% and 15% PEG). The stressed seedlings showed 15.3% and 46.0% lower RWC, respectively, compared to the control group. However, RWC was enhanced by the application of Rut or Si alone and in combination under both stress conditions compared to their respective stress levels. The application of Rut and Si together enhanced RWC by 12.9% under moderate osmotic stress. Furthermore, the application of Rut and Si in combination enhanced RWC by 11.5% under severe stress. The combined treatment with Rut and Si significantly improved RWC under osmotic stress, especially under moderate stress (Fig. 1A).

### TBARS level

Both levels of osmotic stress increased TBARS content, resulting in 80.9% and 162.1% higher levels than the control, respectively. On the other hand, the treatment with Rut + Si resulted in a greater reduction of TBARS content compared to using Rut or Si alone under both stress levels. The reduction effect on TBARS content was significant in seedlings exposed to moderate stress (Fig. 1B).

### H_2_O_2_ level

Under osmotic stress, there was a considerable increase in H_2_O_2_ accumulation. Seedlings subjected to 10% or 15% PEG resulted in 159.7% or 190.3% higher H_2_O_2_ content than the control. However, when Rut + Si were applied in combination, there was a reduction in H_2_O_2_ content by 53.9% and 33.8% in plants exposed to 10% and 15% PEG, respectively, compared to their stress groups (Fig. 1C).

### Osmolyte contents

**Fig. 2:**
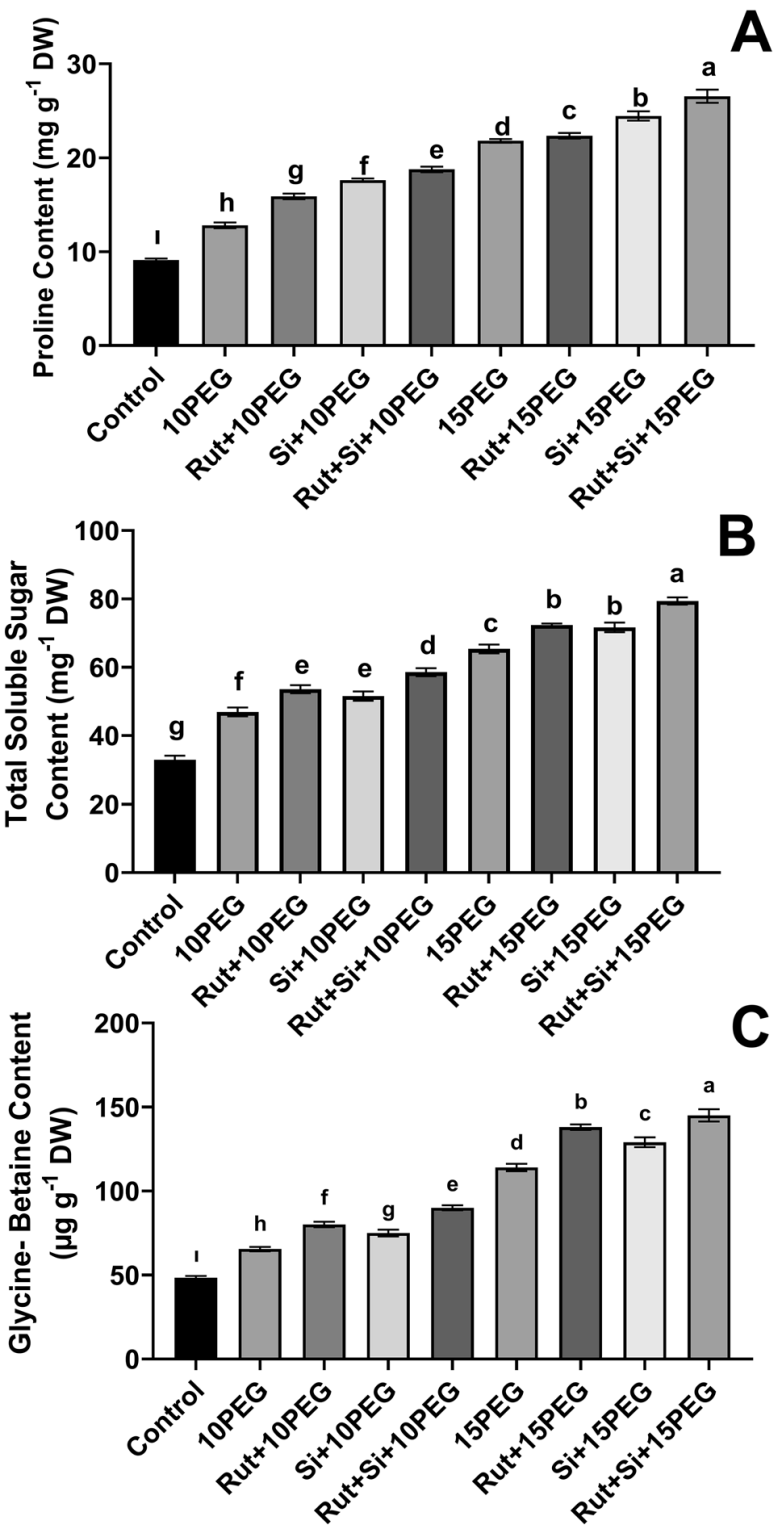
Effect of Rut and Si on Proline content (**A**), Total soluble sugar content (**B**), Glycine-Betaine content (**C**) of maize seedlings under osmotic stress. Vertical bars represent standard deviations. Columns sharing the same letter indicate no significant difference at the 5% level

Compared to the control group, remarkable increases in proline accumulation of 40.7% and 139.6% were recorded in seedlings exposed to 10% and 15% PEG, respectively. Proline accumulation was further enhanced by the exogenous application Rut and Si under both moderate and severe stresses, compared to the same level of stress without Rut and Si in comparison with the same level of stress alone. Additionally, the combined application of Rut + Si resulted in 46.6% and 21.8% higher proline accumulation in plants subjected to 10% and 15% PEG, respectively, compared to the stress groups without Rut and Si (Fig. 2A).

Total soluble sugar content in seedlings exposed to 10% and 15% PEG was 13.9% and 32.4% higher than the control, respectively. Moreover, in seedlings treated with Rut + Si, the total soluble sugar content was 24.9% higher under moderate osmotic stress and 21.5% higher under severe osmotic stress, compared to the respective stress levels without Rut and Si (Fig. 2B).

Compared to the control group, both levels of osmotic stress gave rise to a significant increase in glycine-betaine content, with rises of 35.3% and 135.7% in the plants, respectively. Furthermore, stressed seedlings treated with Rut and Si showed higher glycine-betaine content compared to the seedlings exposed to osmotic stress alone. Additionally, the accumulation of glycine-betaine was more enhanced by the treatment of Rut + Si compared to Rut or Si alone under both stress levels (Fig. 2C).

### Changes in antioxidant capacity

**Fig. 3:**
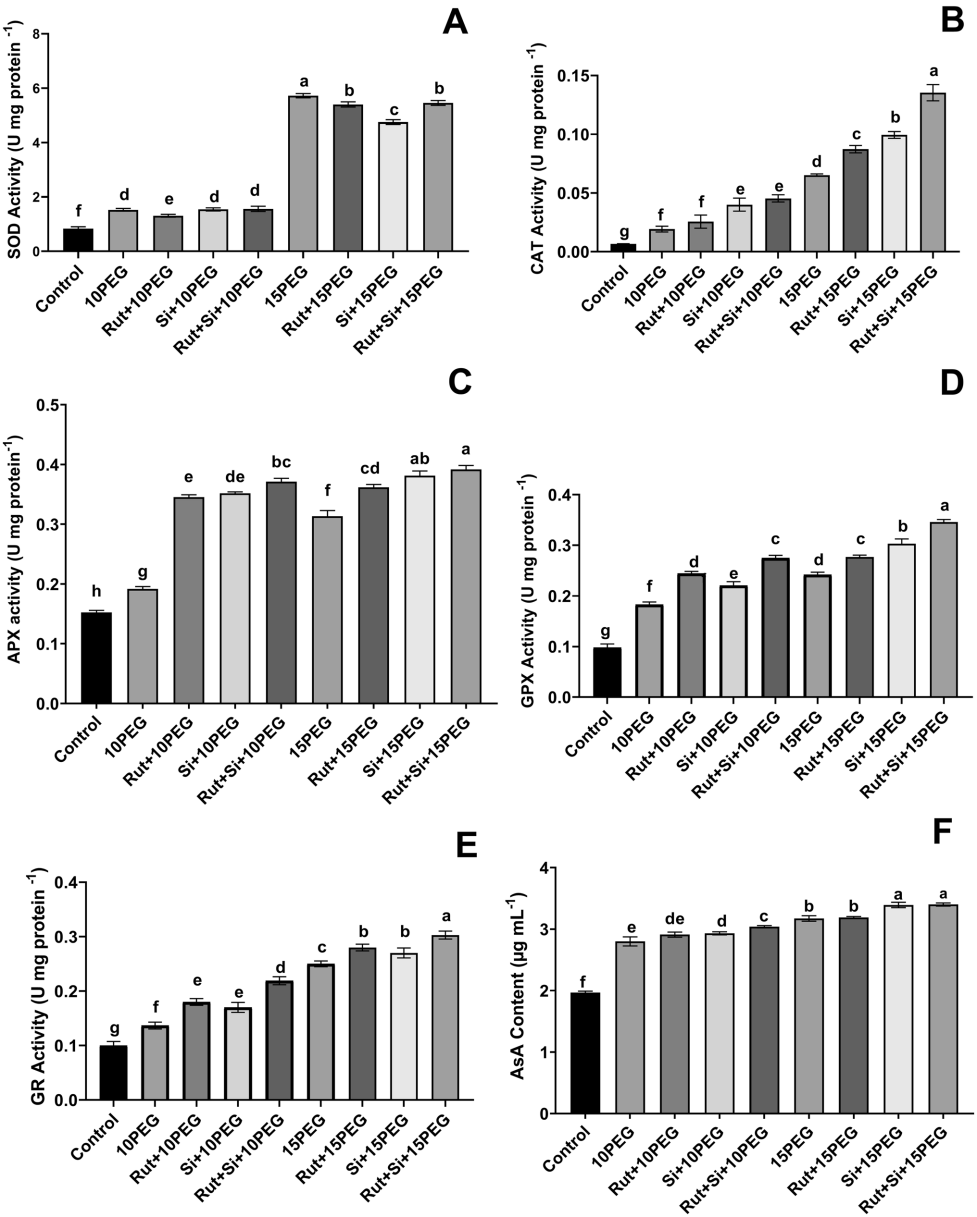
Effect of Rut and Si on SOD activity (**A**), CAT activity (**B**), APX activity (**C**), GPX activity (**D**), GR activity (**E**), AsA content (**F**) of maize seedlings under osmotic stress. Vertical bars represent standard deviations. Columns sharing the same letter indicate no significant difference at the 5% level

Both levels of stress increased the SOD, CAT, APX, and GPX activities as well as the AsA content in maize seedlings (Fig. 3).

Under moderate osmotic stress, Rut application resulted in a decrease in SOD activity compared to moderate osmotic stress alone. The SOD activity in seedlings exposed to moderate osmotic stress was not significantly different from that in seedlings treated with Si or Rut + Si. Under severe osmotic stress, the application of Rut and Si alone as well as in combination decreased the SOD activity compared to severe osmotic stress alone (Fig. 3A).

CAT activity was unaffected in the case of Rut application under moderate osmotic stress, while it increased by 107.5% and 135.2% in seedlings exposed to Si and the Rut + Si combination, respectively, compared to the stress groups without Rut or Si. Additionally, CAT activities in seedlings treated with Rut and Si alone and the Rut + Si combination under severe osmotic stress were 33.9%, 52.5%, and 107.6% higher, respectively, than the same level of stress without Rut or Si (Fig. 3B).

Rut, Si, and Rut + Si increased the APX activity under both osmotic stress levels. Moreover, the exogenous applications of Rut, Si, and Rut + Si significantly enhanced APX activity by 80.2%, 83.4%, and 83.4%, respectively, under moderate osmotic stress compared to the same stress level without Rut or Si (Fig. 3C).

Exogenous treatments of Rut and Si increased GPX activity by 33.6% and 20.6% under moderate osmotic stress, and by 14.5% and 25.3% under severe osmotic stress compared to the osmotic stress groups without Rut or Si. Furthermore, seedlings treated with the Rut + Si combination exhibited an even more significant increase in GPX activity of 50.4% and 43.0% under stress conditions (Fig. 3D).

The GR activity in seedlings exposed to 10% and 15% PEG was 26.8% and 60.0% higher than that in the control. Under moderate osmotic stress, the application of Rut + Si increased GR activity by 37.6% compared to moderate osmotic alone. Moreover, the exogenous application of Rut + Si significantly enhanced GR activity by 17.5% under severe osmotic stress compared to the same stress level without Rut or Si (Fig. 3E).

Exogenously Rut + Si treated seedlings exhibited an 8.5% increase in AsA content under moderate osmotic stress. Furthermore, the AsA content did not show any difference between seedlings treated with Rut and those treated with 15% PEG alone (Fig. 3F).

### Gene expression analysis

**Fig. 4:**
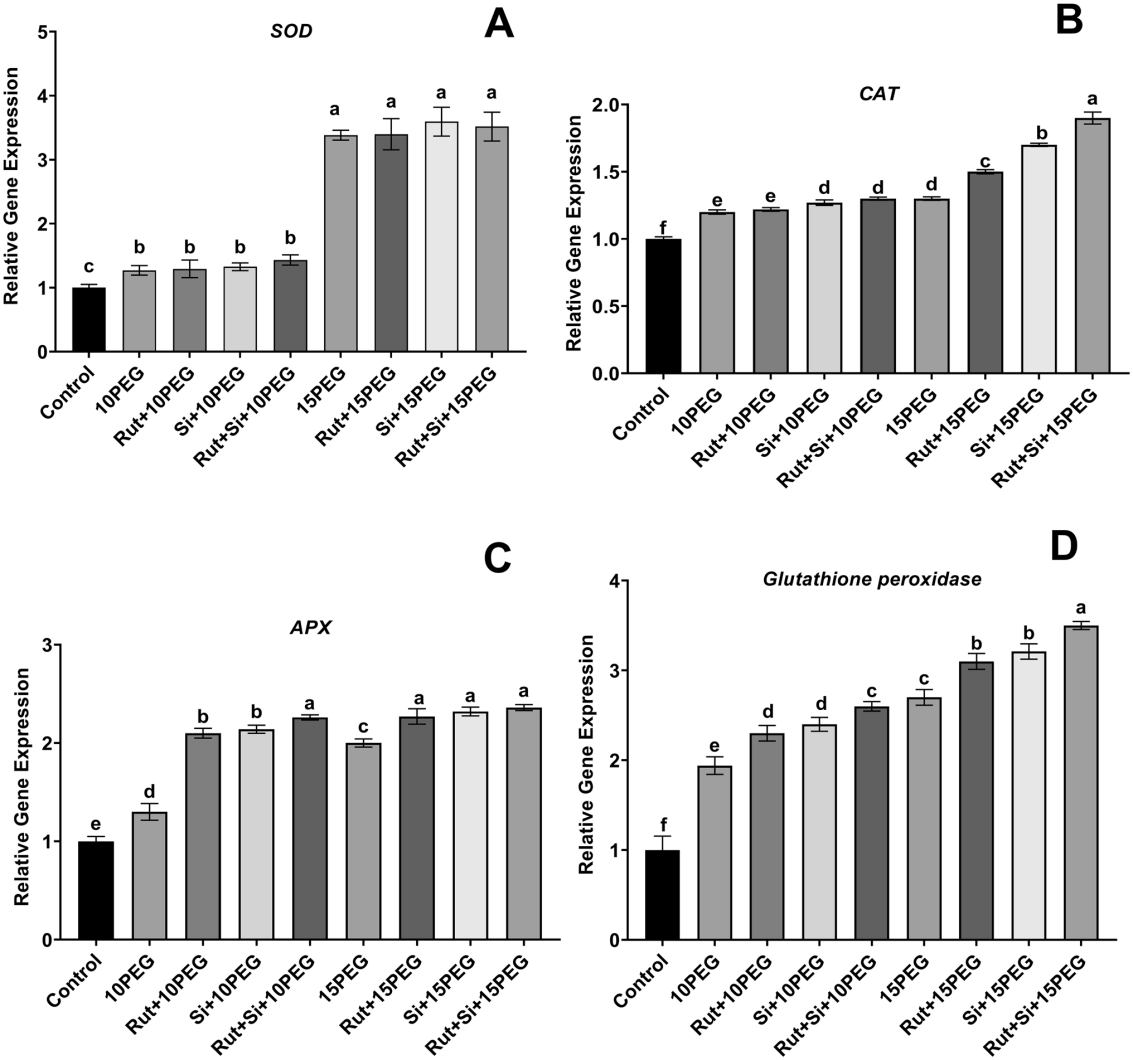
Effect of Rut and Si on relative expression level of *SOD* (**A**), *CAT* (**B**), *APX* (**C**), *Glutathione peroxidase* (**D**) of maize seedlings under osmotic stress. Vertical bars represent standard deviations. Columns sharing the same letter indicate no significant difference at the 5% level

The levels of *SOD*, *CAT*, *APX*, and *glutathione peroxidase* gene expression were determined (Fig. 4). *SOD* expression levels were 1.3 and 3.4 times greater in seedlings treated with 10% and 15% PEG, respectively, than those in the control group. Furthermore, the level of *SOD* expression in seedlings subjected to 10% and 15% PEG was not substantially different from that in seedlings treated to Rut, Si, or Rut + Si under the same osmotic stress (Fig. 4A).

Both levels of osmotic stress caused an increase in the expression level of *CAT*, which was 1.2 and 1.3 times higher in the stressed seedlings compared to the controls. Exogenous treatments of Si doubled the expression level of *CAT* under moderate osmotic stress. Under severe osmotic stress, Rut, Si, and Rut + Si increased the expression level of *CAT* 1.1-, 1.3-, and 1.5-fold, respectively, as compared to severe osmotic stress alone (Fig. 4B).

Compared to the control group, the expression levels of *APX* were upregulated by 10% and 15% PEG, resulting in 30% or 100% higher levels. In addition, exogenous application of Rut + Si significantly increased the expression levels of *APX* by 73.8% under moderate osmotic stress compared to the same stress level alone. Under severe osmotic stress, Rut + Si application increased the expression levels of *APX* by 18.0% compared to severe osmotic stress alone (Fig. 4C).

Under both levels of osmotic stress, the *glutathione peroxidase* expression level was upregulated. In the seedlings under 10% and 15% PEG, respectively, compared to the controls, the increase in the expression level of *glutathione peroxidase* was 1.9 and 2.7 times higher. Under moderate osmotic stress, the expression levels of *glutathione peroxidase* in maize seedlings treated with Rut and Rut + Si were 2.3 and 2.6 times higher than under moderate osmotic stress alone, respectively. Under severe osmotic stress, it was 3.1 and 3.5 times higher than under severe osmotic stress alone (Fig. 4D).

### Correlation of physiological parameters with applications in *Z. mays*

The association between all parameters of *Z. mays* and the applications was depicted in a Pearson correlation table (Table 2). The TBARS level indicated a strong positive correlation with H_2_O_2_ level (r = 0.915^**^). The osmolytes exhibited a positive correlation, with proline showing a strong correlation with both total soluble sugar (r = 0.982^**^) and glycine-betaine (r = 0.959^**^). A remarkable positive correlation was also determined between enzyme activities and related gene expression. However, the leaf RWC was negatively correlated with all the measured parameters.

**Table 2:** Pearson correlation coefficients between applications and all the parameters of *Z.* *mays*

	RWC	TBARS	H_2_O_2_	Proline	Sugar	GB	SOD	CAT	APX	GPX	GR	AsA	*SOD* gene	*CAT* gene	*APX* gene	*Glutathione peroxidase* gene
RWC	1															
TBARS	− 0.970^**^	1														
H_2_O_2_	− 0.851^**^	0.915^**^	1													
Proline	− 0.773^**^	0.686^**^	0.415^*^	1												
Sugar	− 0.779^**^	0.712^**^	0.480^*^	0.982^**^	1											
GB	− 0.825^**^	0.740^**^	0.485^*^	0.959^**^	0.982^**^	1										
SOD	− 0.932^**^	0.902^**^	0.679^**^	0.859^**^	0.872^**^	0.924^**^	1									
CAT	− 0.738^**^	0.651^**^	0.365	0.948^**^	0.943^**^	0.955^**^	0.862^**^	1								
APX	− 0.429^*^	0.292	0.054	0.848^**^	0.816^**^	0.744^**^	0.515^**^	0.708^**^	1							
GPX	− 0.599^**^	0.500^**^	0.286	0.937^**^	0.946^**^	0.879^**^	0.672^**^	0.868^**^	0.908^**^	1						
GR	− 0.784^**^	0.700^**^	0.440^*^	0.978^**^	0.990^*^	0.986^**^	0.887^**^	0.938^**^	0.811^**^	0.922^**^	1					
AsA	− 0.734^**^	0.664^**^	0.513^**^	0.920^**^	0.923^**^	0.850^**^	0.723^**^	0.801^**^	0.856^**^	0.935^**^	0.883^**^	1				
*SOD* gene	− 0.895^**^	0.858^**^	0.608^**^	0.874^**^	0.880^**^	0.931^**^	0.974^**^	0.883^**^	0.564^**^	0.702^**^	0.890^**^	0.752^**^	1			
*CAT* gene	− 0.644^**^	0.557^**^	0.302	0.906^**^	0.906^**^	0.894^**^	0.749^**^	0.963^**^	0.720^**^	0.885^**^	0.874^**^	0.822^**^	0.811^**^	1		
*APX* gene	− 0.473^*^	0.342	0.115	0.859^**^	0.833^**^	0.763^**^	0.549^**^	0.711^**^	0.995^**^	0.909^**^	0.827^**^	0.881^**^	0.596^**^	0.720^**^	1	
*Glutathione peroxidase* gene	− 0.720^**^	0.626^**^	0.410^*^	0.965^**^	0.968^**^	0.926^**^	0.780^**^	0.897^**^	0.885^**^	0.959^**^	0.943^**^	0.968^**^	0.817^**^	0.903^**^	0.905^**^	1

**Correlation is significant at the 0.01 level

*Correlation is significant at the 0.05 level

## Discussion

Numerous environmental stressors, including heat, salinity, and drought, have an impact on plant growth and yield in different crops. The most detrimental kind of stress to plants' physiological and biochemical characteristics among these variables is drought (Tayyab et al. 2020; Naz et al. 2021). Drought stress increased the lipid peroxidation and reactive oxygen species as indicators of drought stress, while it decreased relative water content, photosynthetic pigments, and protein content. Plants were reported to induce their osmoprotectants, phenolics accumulation, and antioxidant enzyme activities in response to drought stress (Khaleghi et al. 2019; Azmat et al. 2020).

Drought is defined as a lack of available water for plant absorption. The primary impact of drought stress on plants is a reduction in water content. This reduction in water content causes growth and physiological disturbances (Ouzounidou et al. 2016). In our study, under osmotic stress conditions, the maize seedlings showed a decrease in leaf relative content with a high proline accumulation demonstrating the presence of drought in plant cells, and these findings are comparable to those of previous research (Saud et al. 2014; Ouzounidou et al. 2016). An increase in RWC was recorded in Si-applied rapeseed seedlings (Hasanuzzaman et al. 2018). This finding is consistent with the findings of the current investigation, in which maize seedlings supplemented with Si under osmotic stress increased leaf RWC compared to osmotic stress alone.

Some studies showed that drought stress enhanced ROS production, such as H_2_O_2_ and O_2_−, causing protein and lipid damage and thereby disrupting membrane stability (Liu et al. 2013; Ye et al. 2016). The increased H_2_O_2_ generation and lipid peroxidation (a higher level of TBARS) in the maize seedlings in the current study indicate membrane damage caused by osmotic stress. After exogenous Rut, Si, and Rut + Si applications to osmotic-stressed seedlings, oxidative stress was alleviated. The findings clearly showed a decrease in the TBARS and H_2_O_2_ contents due to Rut, Si, and Rut + Si with osmotic stress. Previously, it was reported that after Si with drought application TBARS and H_2_O_2_ levels were reduced in grape plants and rapeseed seedlings (Haddad and Kamangar 2015; Hasanuzzaman et al. 2018). Similar to our findings, quercetin, a chemical derivative of Rut (Zeng et al. 2020), significantly decreased the amounts of H_2_O_2_ and TBARS in salt-stressed tomato (Parvin et al. 2019). Moreover, it was found that Si application in drought-stressed wheat cultivars reduced oxidative damage by increasing RWC, cell membrane stability, and the photosynthesis rate (Maghsoudi et al. 2016). In our study, it was clearly demonstrated that Rut is a stress-alleviating compound.

Drought stress results in accumulation of osmolytes (proline, soluble sugar, and glycine-betaine) (Wang et al. 2019). In the current study, the osmotic-stressed maize seedlings showed increases in the levels of osmolytes (proline, soluble sugar, glycine-betaine). Moreover, Rut or Si or Rut + Si caused an extensively increased level of osmolytes. Therefore, the rises in osmolytes caused by Rut, Si, and Rut + Si may contribute to the protection of maize seedlings under both levels of osmotic stress. Exogenous Si treatment has also been reported to improve drought tolerance via the accumulation of osmolytes such as proline and soluble sugar (Parveen et al. 2019; Bukhari et al. 2020). In addition, quercetin, a chemical derivative of Rut (Zeng et al. 2020), was applied to salt-stressed tomato seedlings, and the proline level increased as the amount of quercetin increased (Parvin et al. 2019). Gorni (2022) determined that the total soluble sugar content was increased by Rut application to tomato seedlings grown in the greenhouse. Furthermore, it was reported that Si decreased the glycine-betaine content in drought-stressed corn plants (Parveen et al. 2019), while in another study Si increased the glycine-betaine content in maize plants (Ghasemi et al. 2022). Osmolytes play a positive role in reducing the negative effects of osmotic stress on maize seedlings; this effect of osmolytes may be due to their function in safeguarding the membranes, enzymes, and protein structures that enable seedlings to withstand osmotic stress. In addition, in the present study it was revealed that proline, an osmolyte, plays a more crucial function in stress tolerance when compared to other osmolytes.

SOD, CAT, APX, GPX, GR, etc., enzymatic antioxidants, and ascorbic acid, a nonenzymatic antioxidant, increase to scavenge ROS in stressed plants and protect the cells from oxidative stress (Laxa et al. 2019). SOD is the first line of defense against oxidative damage in cells, and it is involved in the conversion of O_2_^_^ radicals to H_2_O_2_ and oxygen. CAT is involved in the conversion of H_2_O_2_ to H_2_O and oxygen, and it is important in plant metabolism and signal detection (Chung et al., 2017). In our investigation, the activities of SOD and CAT in maize seedlings were significantly higher under both levels of osmotic stress compared to the control treatment. This increase could be due to the fact that SOD and CAT are involved in stress tolerance. Low content of H_2_O_2_ in seedlings treated with Rut or Si or Rut + Si is partly the outcome of increased CAT activity compared to under osmotic stress. Our research demonstrated the beneficial effects of Rut, Si, or Rut + Si on osmotic-stressed maize seedlings in comparison to the control. Under moderate and severe stress, APX activity increased in seedlings compared to the control. The increase in these activities was significantly enhanced by Rut, Si, and Rut + Si applications under osmotic stress compared with the same level of stress alone. Si-applied *Brassica napus* L. plants showed high CAT, APX, and GR activities (Hasanuzzaman et al. 2018). Exogenous Rut, Si, and Rut + Si applications with osmotic stress further enhanced GPX, another important enzyme to catalyze the reaction of H_2_O_2_ scavenging, which increased in osmotic stress-affected maize seedlings, drawing a parallel with the decreased levels of H_2_O_2_ and reduced oxidative damage. Here, we emphasize that CAT and APX may have an active role in reducing H_2_O_2_ level. GR is another important antioxidant enzyme, maintaining cellular redox balance (Dwivedi et al. 2020). In our study, in osmotic stress-exposed maize seedlings, Rut, Si, and Rut + Si applications significantly increased GR activity. Increased GR activity catalyzed the reduction of glutathione disulfide to glutathione (GSH), indicating that GSH level may play an important role in the redox state and protection of cells against ROS. In the literature, it was shown that the SOD, CAT, APX, and GR activities in seedlings exposed to drought stress were induced by Si application, alleviating oxidative damage (Xu et al. 2022). In addition, both soil and foliar Si applications have been reported to increase the activity of antioxidant enzymes in rice compared to arsenic applications alone or, in most cases, even more than the control (Dwivedi et al. 2020). The differences in antioxidant enzyme activities matched the results for gene expression. Our findings demonstrated that the expression levels of *SOD*, *CAT*, *APX*, and *glutathione peroxidase* genes were upregulated by Rut, Si, and Rut + Si applications under osmotic stress. Si treatment has been shown to reduce oxidative damage in some plants by increasing the activity of important antioxidant enzymes like SOD, CAT, GPX, and APX, which aid in the removal of ROS (Parveen et al. 2019; Thorne et al. 2020). Si’s significant influence could be attributed to its role in stimulating enzymes and enhancing the electron transport chain (Kaur and Asthir 2015; Ghaffaria et al. 2021). Furthermore, the crucial role of Rut may be due to its beneficial action as an antioxidant in protecting plant cells from oxidative stress by antioxidant enzyme balance and reduced lipid peroxidation (Yang et al. 2008). It was found that there was excessive AsA accumulation under both stress levels. Additionally, an increasing trend in the AsA content was observed in Rut, Si, and especially Rut + Si supplemented seedlings. AsA, the most effective ROS scavenger, thus could help to reduce H_2_O_2_ overaccumulation and oxidative damage in plants under both stress levels. Our result is in line with the findings indicating that Si application affects the AsA level (Sayed and Gadallah 2014), which is similarly involved in the elimination of ROS.

## Conclusion

The current study showed that exogenous Rut, Si, and Rut + Si applications alleviated the damage caused by osmotic stress in maize seedlings by activating the enzymes of the antioxidant system enzymes, especially CAT, APX, and GR and AsA levels; improved water status; and increased osmolytes. The impact of osmolytes, especially proline, on osmotic stress tolerance might be due to their role in protecting enzymes, protein structures, and membranes that help seedlings tolerate osmotic stress. As a result, osmotically stressed maize seedlings were protected from oxidative harm by reducing the formation of ROS such TBARS and H_2_O_2_. According to the findings, the simultaneous use of Rut and Si proved to be more efficacious in mitigating the adverse impacts of both osmotic stress levels. It is clearly seen that Rut plays an important role in the prominent effects of combined applications in relieving stress. Moreover, this effect was greater in seedlings under moderate osmotic stress. Our study focused on understanding the principal mechanisms involved in Rut + Si-mediated alleviation of osmotic stress tolerance. Moreover, the study may contribute to the elimination of the deficiencies in the literature on the effects of Rut on stress tolerance. It may help researchers plan sustainable agriculture by harnessing Rut- and Si-derived benefits. As evidence of osmotic stress mitigation by Rut, Si, and Rut + Si, the results of this study will encourage further research to shed light on the underlying physiological and molecular mechanisms responsible for alleviating Rut + Si-mediated osmotic stress tolerance in plants.
